# Platelets in preeclamptic pregnancies fail to exhibit the decrease in mitochondrial oxygen consumption rate seen in normal pregnancies

**DOI:** 10.1042/BSR20180286

**Published:** 2018-05-08

**Authors:** Andrew M. Malinow, Rosemary A. Schuh, Omar Alyamani, Joseph Kim, Shobana Bharadwaj, Sarah D. Crimmins, Jessica L. Galey, Gary Fiskum, Brian M. Polster

**Affiliations:** 1 Department of Anesthesiology, University of Maryland School of Medicine, S11D15-UMMC, 22 S. Greene St, Baltimore, MD 21201, U.S.A.; 2 Department of Obstetrics, Gynecology and Reproductive Sciences, University of Maryland School of Medicine, S6D-UMMC, 22 S. Greene St, Baltimore, MD 21201, U.S.A.

**Keywords:** mitichondria, platelets, pregnancy, preeclampsia

## Abstract

Cellular oxygen consumption and lactate production rates have been measured in both placental and myometrial cells to study obstetrics-related disease states such as preeclampsia. Platelet metabolic alterations indicate systemic bioenergetic changes that can be useful as disease biomarkers. We tested the hypothesis that platelet mitochondria display functional alterations in preeclampsia. Platelets were harvested from women in the third trimester of either a healthy, non-preeclamptic or preeclamptic pregnancy, and from healthy, non-pregnant women. Using Seahorse respirometry, we analyzed platelets for oxygen consumption (OCR) and extracellular acidification (ECAR) rates, indicators of mitochondrial electron transport and glucose metabolism, respectively. There was a 37% decrease in the maximal respiratory capacity measured in platelets from healthy, non-preeclamptic compared with preeclamptic pregnancy (*P*<0.01); this relationship held true for other measurements of OCR, including basal respiration; ATP-linked respiration; respiratory control ratio (RCR); and spare respiratory capacity. RCR, a measure of mitochondrial efficiency, was significantly lower in healthy pregnant compared with non-pregnant women. In contrast with increased OCR, basal ECAR was significantly reduced in platelets from preeclamptic pregnancies compared with either normal pregnancies (−25%; *P*<0.05) or non-pregnant women (−22%; *P*<0.01). Secondary analysis of OCR revealed reduced basal and maximal platelet respiration in normal pregnancy prior to 34 weeks’ estimated gestational age (EGA) compared with the non-pregnant state; these differences disappeared after 34 weeks. Taken together, findings suggest that in preeclampsia, there exists either a loss or early (before the third trimester) reversal of a normal biologic mechanism of platelet mitochondrial respiratory reduction associated with normal pregnancy.

## Introduction

In normal pregnancy, placental mitochondria produce excessive reactive oxygen species [1]. Imbalance of antioxidant capacity can result in oxidative damage [1]. Preeclampsia, a leading cause of maternal and fetal/neonatal morbidity and mortality [2–4], is characterized by abnormal placentation (imbalance in pro- and anti-angiogenic factors), an inflammatory response with accompanying immunologic dysfunction, placental and maternal systemic endothelial dysfunction, and altered coagulation [5]. Mitochondria within placental [6–10] and myometrial [11] cells harvested at delivery in pregnancies afflicted by preeclampsia demonstrate various histologic (e.g. distorted inner membranes) and functional changes (i.e. reduced or increased respiration, desensitization to calcium depolarization) as compared with normal pregnancies.

Platelets have limited circulation lifetimes [12], have limited mitophagy (a major mechanism for the removal of damaged mitochondria), and do not produce new mitochondria [13]. Though platelets have few mitochondria per cell, established techniques for isolation of a large number of platelets provide a sample with a relatively high mitochondrial content for respirometric analysis. Therefore, platelets are a sensitive indicator of changes in mitochondrial function [14,15], consistent with their deficiency in mitochondrial turnover. In preeclamptic pregnancy, platelets are reported to exhibit increased oxidative protein carbonyl modifications and decreased antioxidant catalase enzyme activity compared with platelets in normal pregnancy [16]. Because oxidative stress can impair cellular bioenergetics [17], we hypothesized that platelets harvested from preeclamptic pregnancy exhibit bioenergetic dysfunction relative to platelets harvested from either the non-preeclamptic pregnancy or the non-pregnant state. To test this hypothesis, we compared three groups: non-pregnant healthy women; healthy, pregnant women in third trimester of gestation not diagnosed with preeclampsia; and, women in third trimester of gestation diagnosed with preeclampsia. Platelets were collected and analyzed for mitochondrial oxygen consumption and extracellular acidification, an indicator of glycolysis.

## Materials and methods

### Human volunteers

In all, 64 women were approached about participation in the study; 60 agreed to participate, all were screened for inclusion into the study. After the study protocol was explained, patients gave written informed consents to a protocol approved by the institutional Human Research Protection Office. All study participants were 18–40 years old.

Twenty-one healthy, non-pregnant volunteers were screened to serve as non-pregnant controls and, following one refusal, twenty were enrolled into the study. Study participants in the non-pregnant group were nulliparae, who denied a (previous or present) history of hypertension or diabetes (see Table 1).

**Table 1:** Demographic and clinical characteristics of study patients

Characteristics	Non-pregnant (*n*=19)		Healthy pregnant (*n*=20)		Preeclamptic pregnant (*n*=20)		*P*-value
	Mean ± S.D.	Range	Mean ± S.D.	Range	Mean ± S.D.	Range	
Age (years)	28.50 (± 2.48)	22–32	27.25 (± 4.36)	21–35	27.05 (± 6.13)	19–39	0.775
Race/ethnicity							
NHW	14		2		6		
EI	2		0		0		
AA	1		17		11		
AS	2		0		1		
HS	1		0		2		
AF	0		1		0		
Gravidity	0	0	3.15 (± 2.83)	1–14	2.80 (± 2.48)	1–11	0.680
Parity	0	0	1.15 (± 0.99)	0–3	0.55 (± 0.95)	0–4	0.057
EGA (weeks)			36^1/7^(± 4.6)		32^1/7^(± 3.56)		0.005

Abbreviations: AA, African American; AF, African; AS, Asian; EGA, estimated gestational age; EI, East Indian; HS, Hispanic; NHW, non-Hispanic white.

Data are presented as mean ± S.D. One-way ANOVA was used to determine intergroup differences for age. Gravidity, parity, and EGA gestational age were examined by *t*test for direct comparison between Healthy pregnant and Preeclamptic pregnant.

Forty-three pregnant women presenting to Labor and Delivery at our academic teaching hospital as well as those cared for at antenatal clinics staffed by faculty members of our medical school were also screened and then approached for enrollment into the study. All patients were in the third trimester of pregnancy. Three patients refused; forty patients were enrolled in two cohorts (see Table 1).

The first group of pregnant patients was designated as Healthy pregnancy. Patients were at least 26 weeks’ estimated gestational age (EGA) who had not been diagnosed with preterm labor, preeclampsia or hypertension, diabetes, intrauterine growth restriction (IUGR) and were receiving only routine antenatal care (see Table 1). The second group of patients was designated as Preeclamptic pregnancy. Only 1 of the 20 preeclamptic pregnancies was diagnosed with HELLP syndrome, a pregnancy complication consisting of hemolysis, elevated liver enzymes, and low platelet count. Her platelet count decreased from 67000 to 37000 per μl over a 4-h period during which the study sample was drawn, having little impact on the mean platelet count of the group. Patients were at least 26 weeks’ EGA and all were diagnosed with preeclampsia using current criteria promulgated by the American College of Obstetrics and Gynecology) [18] (see Table 1).

Peripheral venipuncture was performed to collect 7–10 ml aliquots of whole blood in vacutainers (BD, Franklin Lakes, NJ) with dipotassium EDTA as an anticoagulant. All patients were identified by a study number, de-identifying the subject (from their name and study group) to the lone investigator performing laboratory analysis. The study number was written on to a label then affixed to the tube. The tube was carried immediately to the site of laboratory analysis in another building on-campus.

### Chemicals

All chemicals were purchased from Sigma-Aldrich (St. Louis, MO) unless otherwise stated.

### Platelet isolation

Isolations were performed at room temperature using anticoagulants to prevent cell activation during the isolation procedure. Whole blood was spun at 500×***g*** for 10 min at 25°C with soft acceleration/deceleration using a Sorvall ST 8R centrifuge (Thermo Scientific, Rockford, IL) to separate the platelet rich plasma which was transferred to 15 ml conical polypropylene centrifuge tubes (Thermo Scientific) under aseptic conditions. Following removal of aliquots to be frozen at −80°C for further processing, the platelet rich plasma was spun at 1600×***g*** for 10 min at 25°C to separate the platelet poor plasma and platelets. The platelet poor plasma was removed and frozen at −80°C for later use. The platelet pellets were resuspended in pre-warmed (37°C) assay measurement buffer (Dulbecco’s modified Eagle’s medium supplemented with 1 mM pyruvate, 6.2 mM EDTA, 5.5 mM d-glucose, and 4 mM l-glutamine, pH 7.4).

### Cell counts

Cell counts from platelet poor plasma, platelet rich plasma, and platelets in measurement buffer were acquired using a Beckman Z1 Particle Counter (Beckman Coulter, Brea, CA). Size restrictions were set to measure particles between 1.8 and 3.9 µm and measurements were done according to the manufacturer’s protocol.

### Platelet attachment for bioenergetics studies

The platelets were attached to V7 polystyrene plates (Agilent Technologies, Santa Clara, CA) according to a method modified from Chacko et al. [19]. Specifically, platelets were diluted in assay measurement buffer to yield 60 million cells/well. Platelets were seeded in a 200-μl volume on to plates pre-coated with the cell adhesive Cell-Tak (Corning, Bedford, MA). The plates were placed in a non-CO_2_ incubator at 37°C for 30 min to sediment. Following sedimentation, the platelets were spun at 1600×***g*** for 5 min at 25°C with soft acceleration/deceleration, then the plates were rotated at 180° and the spin was repeated. A further 475 µl assay measurement buffer was added to the wells and the plates were placed in a non-CO_2_ incubator at 37°C for 45 min to equilibrate prior to bioenergetic measurements. A minimum of three replicate wells per sample were used for each experiment. Bioenergetic measurements were initiated for all samples within 180–240 min after venipuncture.

### Platelet bioenergetic measurements

Following three OCR (basal oxygen consumption rate) measurements, oligomycin (2.5 µM), an ATP synthase inhibitor, was injected and two OCR measurements were acquired. To induce maximal respiration, the proton ionophore 2,4-DNP (2,4-dinitrophenol, 15 µM) was injected and two OCR measurements were performed. A second injection of 2,4-DNP was made to ensure that mitochondria were fully uncoupled. After an additional two measurements, antimycin A (10 µM), an inhibitor of Complex III of the electron transport chain, was injected to assess antimycin A-insensitive non-mitochondrial oxygen consumption. Three measurements were acquired and then the experiment was terminated. Optimal concentrations of oligomycin and 2,4-DNP for mitochondrial functional assessment were determined by titration of the compounds in separate pilot experiments. Platelet lysates from all wells were collected in lysis buffer containing radioimmunoprecipitation assay (RIPA) buffer (5 mM Tris/HCl, pH 8.0, 15 mM NaCl, 0.05% deoxycholate, 0.1% NP-40, 0.1% SDS), and protease inhibitor cocktail (EMD Millipore), then immediately stored at −20°C until a Micro BCA assay (Thermo Scientific) was performed to measure protein content. OCR and extracellular acidification rate (ECAR) values were normalized to platelet total protein content.

For analyses, ATP-linked respiration was calculated by subtracting the residual OCR after oligomycin addition from the baseline cellular OCR. Proton leak respiration was approximated as oligomycin-insensitive OCR. Maximal respiratory capacity was considered to be the DNP-induced OCR. Spare respiratory capacity was calculated by subtracting basal OCR from maximal respiratory capacity. Maximal respiratory control ratio (RCR) was calculated as the DNP-induced OCR divided by the oligomycin-induced OCR.

### Statistical analysis

The pre-experimental statistical plan considered results from three groups of 20 patients using ANOVA enough to detect a 15% difference in means and a 15% difference in S.D. with a power = 0.8; α = 0.05. All results were reported as mean ± S.D. Parametric statistical analysis was performed using SigmaPlot version 12.0 (SYSTAT Software, San Jose, CA). Rates of mitochondrial respiration were compared by one-way ANOVA with Holm–Sidak *post hoc* test to determine intergroup differences. Direct comparisons between two groups were compared by *t* test with multiple linear regression analyses to control for demographic and clinical characteristics; *P*-values <0.05 were considered significant. Graphs were generated in Excel 2010 (Microsoft, Redmond, WA).

## Results

There was no significant difference in age amongst women in any of the three study groups (*P*=0.775 (ANOVA, Table 1). There was no significant difference in gravidity or parity between women in the healthy pregnancy and preeclamptic pregnancy groups (*P*=0.680 and *P*=0.057, respectively) (Table 1). There was no significant difference in the BMI (kg/m^2^) between women in the healthy pregnancy (mean = 37; median = 36; range = 21–57) and preeclamptic (mean = 40; median = 37; range = 28–64) groups (*P*=0.365). There was a statistical difference (*P*<0.01) in the EGA of pregnancies between the healthy pregnancy and preeclamptic pregnancy groups (mean = 36^1/7^ weeks’ and 32^2/7^ weeks’ EGA, respectively) (Table 1).

Amongst women in the preeclamptic pregnancy group, all had been admitted to the hospital, diagnosed with preeclampsia, and subsequently treated with intravenous magnesium sulphate therapy and oral and/or intravenous antihypertensive medication. Magnesium infusion was initiated before the study sample was drawn in all but three of the women in the preeclamptic group soon thereafter in the remainder of the group. Antihypertensive medications administered in 17 women in the preeclamptic group at some time (either earlier in pregnancy or during the current admission) before the study sample was drawn, including, as single- or multiple-dose therapy: labetalol (16 of 17); nifedipine (6 of 17); α-methyldopa (1 of 17); and hydralazine (2 of 17). On the day that the study sample was drawn, maternal systemic blood pressure of the preeclamptic cohort ranged 130–170/75–108 mmHg. Antihypertensive treatment was initiated in the remaining three patients after the study sample was drawn but during their hospital admission. Low-dose aspirin was administered in five patients at some time in the pregnancy before the study sample was drawn: two patients had finished a course of long-term therapy (81 mg per day) at least 1 week prior to venipuncture; three patients were administered single doses of 81 or 162 mg, two at some time during the week before venipuncture. Amongst women in the preeclamptic pregnancy group, the mean platelet sample-to-delivery time was less than 3 days (minimum/median/maximum = 0/3/9).

A blood sample from one woman in the non-pregnant group was deemed unusable due to partial clotting of the blood and was discarded. Venipuncture was not repeated, leaving 19 blood samples in the non-pregnant group that were usable for bioenergetic analysis. All samples drawn from women in both the healthy pregnancy and preeclamptic pregnancy groups were deemed suitable for analysis.

### Platelet rich plasma platelet counts in both healthy and preeclamptic pregnancies

**Figure 1:**
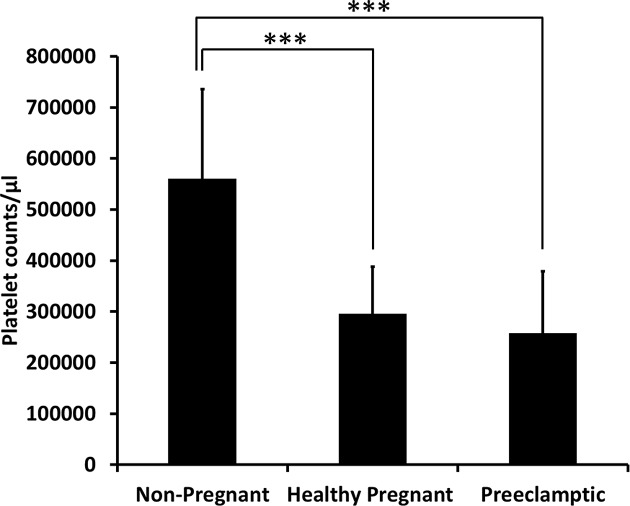
Platelet counts in platelet rich plasma Platelet number per microliter was assessed in platelet rich plasma isolated from whole blood collected from non-pregnant (*n*=19), healthy pregnant (*n*=20), and preeclamptic pregnant (*n*=20) women. Data are presented as mean ± S.D. ***P<0.001.

The distribution of platelet count (per microliter) of the platelet rich plasma amongst the groups was significantly different (*P*<0.001) (Figure 1). The mean platelet count of the platelet rich plasma in the non-pregnant group was significantly higher (approximately double, *P*<0.001) compared with either the healthy pregnancy or preeclamptic pregnancy groups (Figure 1). Inclusion of the one patient with HELLP syndrome (described above) had no significant impact on the mean platelet count of the preeclamptic cohort.

### Basal and maximal OCRs are increased in platelets isolated from preeclamptic pregnancies compared with those from healthy pregnancies

**Figure 2:**
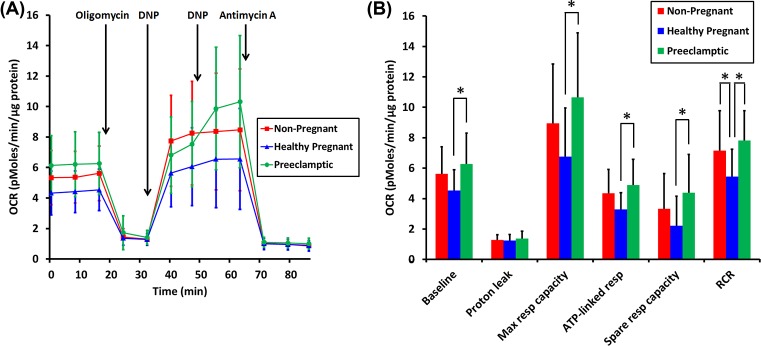
Mitochondrial respiration by isolated platelets (**A**) Average OCR (pmoles/min/µg protein) by platelets isolated from non-pregnant (red squares, *n*=19), healthy pregnant (blue triangles, *n*=20) and preeclamptic pregnant (green circles, *n*=20) women. Oligomycin (2.5 μM), 2,4-DNP (15 μM), and antimycin A (10 μM) were added when indicated. (**B**) Graphical presentation of OCR rates computed from the traces in (A) as described in the ‘Materials and methods’ section. Red lines or bars (non-pregnant), blue lines or bars (Healthy pregnant) and green lines or bars (Preeclamptic pregnant). Data are presented as mean ± S.D. *P<0.05.

To test for potential alterations in mitochondrial bioenergetic properties for healthy pregnancy and preeclamptic pregnancy as compared with non-pregnant volunteers, platelets were isolated from whole blood and mitochondrial function was assessed by cell-based respirometry. Basal OCR was significantly different across groups (*P*=0.010) with an increase (*P*<0.01) in preeclamptic pregnancy as compared with healthy pregnancy (Figure 2). There was no difference between non-pregnant and preeclamptic pregnancy groups (*P*=0.256) or between non-pregnant and healthy pregnancy (*P*=0.114) groups (Figure 2). The ATP synthase inhibitor oligomycin was added to enable estimation of ATP-linked respiration (that proportion of mitochondrial respiration decreased by oligomycin) and residual mitochondrial respiration driven primarily by proton leak. ATP-linked respiration was significantly different across groups (*P*=0.004). The ATP-linked OCR for healthy pregnancy was significantly decreased compared with the preeclamptic pregnancy (*P*<0.01) group; there was a strong trend toward a decrease (*P*=0.056) compared with the non-pregnant group (Figure 2B). There were no significant differences in proton leak across groups (*P*=0.593, Figure 2B). Following addition of the uncoupler DNP to induce maximal respiration, there was a significant difference in OCR across groups (*P*=0.008), with the preeclamptic pregnancy group showing a significant increase (*P*<0.01) relative to the healthy pregnancy group (Figure 2). The mitochondrial spare respiratory capacity (maximal OCR minus basal rate) was also significantly different across groups (*P*=0.014). The preeclamptic pregnancy group displayed significantly increased (*P*<0.05) spare respiratory capacity as compared with healthy pregnancy (Figure 2B). There were no differences in spare respiratory capacity when comparing non-pregnant with either healthy pregnancy (*P*=0.249) or preeclamptic pregnancy groups (*P*=0.147, Figure 2B). There was a significant difference (*P*=0.003) in RCR, defined here as the ratio of maximal mitochondrial OCR induced by DNP addition to mitochondrial OCR measured in the presence of oligomycin. The RCR was significantly decreased for the healthy pregnancy group when compared with either the preeclamptic pregnancy group (*P*<0.01) or to the non-pregnant cohort (*P*<0.05). There was no difference in RCR (*P*=0.341) between the non-pregnant and preeclamptic pregnancy groups (Figure 2B).

Two additions of DNP were sequentially made in all experiments to ensure that the maximal respiration for each platelet sample was achieved. The second DNP addition further stimulated respiration by platelets from preeclamptic patients (*P*<0.001) but not those from the healthy pregnant (*P*=0.452) or non-pregnant cohorts (*P*=0.723).

### ECAR is decreased in platelets isolated from preeclamptic pregnancies

**Figure 3:**
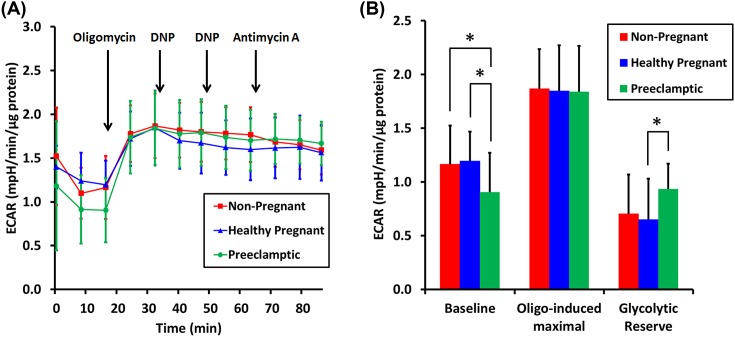
ECARs by isolated platelets (**A**) Average ECAR (mpH/min/µg protein) by platelets isolated from non-pregnant (red squares, *n*=19), healthy pregnant (blue triangles, *n*=20), and preeclamptic pregnant (green circles, *n*=20) women. The measurements were acquired simultaneously with OCRs and platelets received the same drug injections as in Figure 2A. (**B**) Graphical presentation of ECARs computed from the traces in (A) as described in the ‘Results’ section. Red bars (non-pregnant), blue bars (Healthy pregnant), and green bars (Preeclamptic pregnant). Data are presented as mean ± S.D. *P<0.05.

ECAR, an indirect measurement of glycolysis, was also assessed in the platelets from the three different cohorts. Basal ECAR was significantly different across groups (*P*=0.015, Figure 3). There was no significant difference in basal ECAR comparing non-pregnant and healthy pregnancy groups (*P*=0.776) (Figure 3B). However, basal ECAR was significantly decreased (*P*<0.05) in preeclamptic pregnancy when compared with the other two cohorts (Figure 3B). Inhibition of mitochondrial ATP synthesis by oligomycin stimulates glycolysis and was used to estimate the maximal glycolytic capacity and the glycolytic reserve, defined as the difference between basal and oligomycin-induced ECAR. There was no significant difference in oligomcyin-induced ECAR amongst groups (*P*=0.975), suggesting no differences in maximal glycolytic capacity. However, decreased basal ECAR in the preeclamptic pregnancy group resulted in a significant increase (*P*<0.05) in the glycolytic reserve when compared with the healthy pregnancy group but not when compared with the non-pregnant group (*P*=0.067) (Figure 3B).

### Secondary analysis

#### Effect of EGA and IUGR on platelet respiration

**Figure 4:**
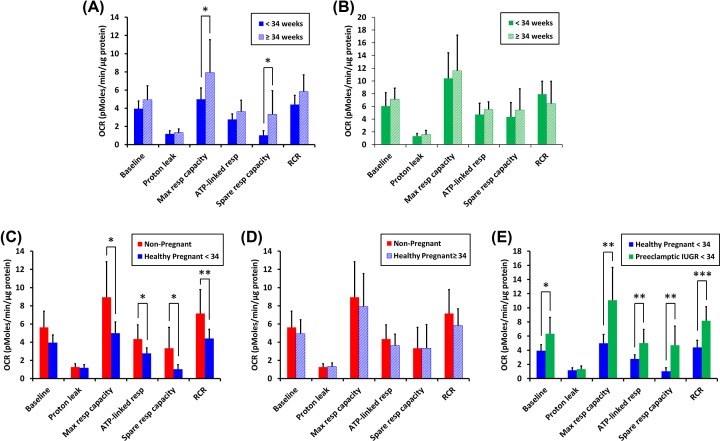
Effect of EGA on respiration by isolated platelets (**A**) Average OCRs (pmoles/min/µg protein) for the experiment described by Figure 2 were compared for platelets isolated from healthy pregnant women <34 weeks EGA (*n*=8, solid blue bars) with those cases ≥34 weeks EGA (*n*=12, striped blue bars). (**B**) The same comparison as in (A) was made for platelets from preeclamptic pregnant <34 weeks EGA (*n*=16, solid green bar) and preeclamptic pregnant ≥34 weeks EGA (*n*=4, striped green bars). (**C**) Average OCRs by isolated platelets from non-pregnant women (red bars) and healthy pregnant women <34 weeks EGA (blue bars) were compared. (**D**) Average OCRs by isolated platelets from non-pregnant women (red bars) and healthy pregnant women ≥34 weeks EGA (blue bars) were compared. (**E**) Average OCRs between healthy pregnant women <34 weeks EGA (*n*=8, blue bars) and preeclamptic cases <34 weeks EGA that also had IUGR (*n*=10, green bars) were compared. Data are presented as mean ± S.D. *P<0.05, **P<0.01, ***P<0.001.

As noted earlier, there was a statistical difference (*P*<0.01) in the EGA of pregnancies between the healthy pregnancy and preeclamptic pregnancy groups at a time when platelets were sampled (See Table 1). The EGA of the majority of the preeclamptic pregnancy group (*n*=16 out of 20) was less than 34 weeks whereas the EGA of the minority of the healthy pregnancy group (*n*=8 out of 20) was less than 34 weeks. In the healthy pregnancy group, there were statistical differences in OCR amongst women whose EGA was less than 34 weeks (<34, *n*=8) and women whose EGA was equal to or greater than 34 weeks (≥34, *n*=12). Maximal and spare respiratory capacity were significantly higher (*P*<0.05) in the healthy pregnancy group with EGA ≥34 weeks compared with healthy pregnant women with EGA <34 weeks (Figure 4A). In the preeclamptic pregnancy group, there was no statistically significant difference in any respiratory parameter comparing pregnancies <34 weeks (*n*=16) and ≥34 weeks EGA (*n*=4) (Figure 4B).

Given the effect of EGA on the platelet bioenergetic properties of the healthy pregnancy cohort, platelets of EGA <34 weeks and of EGA ≥34 weeks from this group were separately compared with the non-pregnant cohort. Basal respiration, maximal respiration, ATP-linked respiration, spare respiratory capacity, and RCR were all significantly lower in the EGA <34 pregnant platelets when compared with platelets from non-pregnant patients (*P*<0.05; *P*<0.01 for RCR) (Figure 4C); for pregnancies with EGA ≥34 weeks, there were no significant differences in any of the mitochondrial bioenergetic parameters (Figure 4D).

Preeclamptic pregnancy patients of EGA <34 weeks that exhibit IUGR are considered to have a severe form of the disease. Comparing platelet mitochondrial bioenergetics of this preeclamptic IUGR population with the platelets from healthy pregnancies at EGA <34 weeks revealed major increases in basal respiration, maximal respiration, ATP-linked respiration, spare respiratory capacity, and RCR for the preeclamptic group (Figure 4E) (*P*<0.05 for basal respiration; *P*<0.01 for maximal respiration, ATP-linked respiration, and spare respiratory capacity; *P*<0.001 for RCR). There were no differences measured in the preeclamptic pregnancy group diagnosed as IUGR (*n*=12) as compared with the preeclamptic pregnancy group not diagnosed as IUGR (*n*=8) (data not shown).

We performed regression analyses to control for EGA and also parity (number of pregnancies carried greater than 20 weeks). Basal, ATP-linked respiration, maximal respiratory capacity, mitochondrial spare capacity, and RCR remained significantly increased (*P*≤0.001) in the preeclamptic group as compared with the healthy pregnant group of women, regardless of EGA or parity effect (Table 2).

**Table 2:** Multivariate regression analysis of associations between OCR and clinical parameters

	Coefficients^1^			
	Unstandardized coefficients			
Model	B	S.E.M.	*t*	*P*-value
**Baseline**				
(Constant)	4.213	0.689	6.110	<0.001
Pregnancy group	2.004	0.625	3.205	0.003
EGA	0.956	0.609	1.570	0.125
Parity	−0.358	0.574	−0.624	0.537
**Maximal respiratory capacity**				
(Constant)	5.387	1.496	3.601	<0.001
Pregnancy group	4.808	1.357	3.544	0.001
EGA group	2.241	1.321	1.697	0.098
Parity	0.0278	1.245	0.0224	0.982
**ATP-linked respiration**				
(Constant)	3.101	0.563	5.507	<0.001
Pregnancy group	1.793	0.511	3.511	0.001
EGA group	0.775	0.497	1.558	0.128
Parity	−0.396	0.469	−0.845	0.404
**Spare respiratory capacity**				
(Constant)	1.174	0.896	1.311	0.198
Pregnancy group	2.804	0.812	3.451	0.001
EGA group	0.775	0.791	1.626	0.113
Parity	0.386	0.746	0.517	0.608
**RCR**				
(Constant)	5.139	0.764	6.730	<0.001
Pregnancy group	2.607	0.692	3.765	<0.001
EGA group	0.783	0.674	1.161	0.253
Parity	−0.227	0.635	−0.357	0.723

^1^Dependent variable: OCR.

## Discussion

Preeclampsia is associated with an increase in platelet activation and consumption [20–22] leading, in more severe cases, to both quantitative deficits (decrease in platelet count [23,24], increase in mean platelet volume [25]) and hemostatic dysfunction as defined by aggregometry [26]. Based on elevated oxidative stress in preeclampsia [10] and evidence that oxidative stress can cause mitochondrial dysfunction [17], we expected that mitochondrial impairments (e.g. reduced RCR) would be observed. In contrast with our expectation but consistent with a unique bioenergetic signature, we found that preeclampsia is associated with significant increases in several bioenergetics parameters compared with healthy pregnancy including: basal respiration; ATP-linked respiration; maximal and spare respiratory capacity; and RCR. RCR is the ratio of maximal respiration to respiration driven by proton leak and is used to estimate maximal efficiency of energy transduction between the redox potential energy released by the electron transport chain and the formation of the high-energy phosphodiester bond present in ATP. These results are consistent with changes previously found in placental [6,8–10,27] and myometrial mitochondria [11], suggesting that platelets may be useful in the antenatal study of mitochondrial bioenergetics profiles in pregnancies afflicted with preeclampsia or other pregnancy-associated disease. Interestingly, none of the respiratory measurements in platelets harvested from preeclamptic pregnancies was significantly different from that observed in platelets from non-pregnant women, suggesting that the changes observed in preeclampsia may represent failure of a normal, possibly adaptive, biologic mechanism initiated during pregnancy.

RCR was significantly decreased in healthy pregnancy as compared with the non-pregnant state. This unexpected finding suggests that under conditions of maximal energy demand, mitochondria from pregnant women will be less efficient in ATP production than mitochondria from non-pregnant women; that is, the amount of oxygen consumption uncoupled from ATP synthesis will be a greater percentage of the total oxygen consumed. A secondary analysis of our data, limited by smaller patient numbers, indicates that in addition to decreased RCR, platelets from healthy pregnancies less than 34 weeks’ EGA have decreased basal, ATP-linked, and maximal mitochondrial respiration as compared with non-pregnant women. These findings might be explained by a pregnancy-associated reduction in platelet mitochondrial number as previously observed by electron microscopy [28]. The reasons for these pregnancy-linked mitochondrial changes are unclear. However, since mitochondria are a major source of cellular reactive oxygen species production [29], it is possible that the decrease in mitochondrial number and function are part of an adaptive mechanism to limit oxidative stress.

There was no change in platelet mitochondrial respiration in preeclamptic pregnancy when comparing pregnancies at <34 weeks’ EGA with those ≥34 weeks’ EGA. However, the platelet bioenergetics profile of healthy pregnant women ≥34 weeks’ EGA demonstrated a reversal of the respiratory changes observed more distant to <34 weeks’ EGA. In fact, measurements were similar to that seen both in non-pregnant and in preeclamptic women. These results suggest that any adaptive mechanism seen in routine pregnancy is either ‘lost’ earlier than could be detected or is totally absent from our group of preeclamptic pregnancies. In addition, we compared platelet bioenergetic profiles of preeclamptic pregnancies of EGA <34 weeks also diagnosed with IUGR to healthy (therefore, not diagnosed with IUGR) pregnancies of EGA <34 weeks. The group of preeclamptic patients with IUGR demonstrate an increase in platelet respiratory capacity (Figure 4E). There are no differences in the results when comparing preeclamptic patients with IUGR to those preeclamptic patients not diagnosed with IUGR. These results suggest that the mechanism underlying altered preeclamptic platelet bioenergetics may be unrelated to the mechanism underlying IUGR. The number of patients included in these secondary analyses is small. Further studies, including larger numbers of patients, will be needed to confirm or reject these results.

Preeclamptic women display lower ECAR than platelets from normal pregnancies or non-pregnant women (Figure 3A,B). ECAR is largely due to glycolytic lactic acid production and carbonic acid formed from tricarboxylic acid cycle-derived carbon dioxide [30]. Because preeclamptic women exhibit greater basal respiration, and therefore greater tricarboxylic acid cycle flux, the decrease in ECAR can be interpreted as a reduced rate of glycolysis. Unchanged glycolytic capacity in preeclamptic platelets (Figure 3B), accompanied by increased OCR (Figure 2B) suggests decreased utilization of aerobic glycolysis and increased utilization of mitochondrial oxidative phosphorylation for ATP production. Perhaps in keeping with this result, we found that a higher concentration of the uncoupler DNP was necessary to elicit maximal respiration from preeclamptic platelets as compared with the concentration required for platelets from healthy pregnant or non-pregnant women. The higher DNP requirement could be due to increased mitochondrial mass or inner membrane surface area, consistent with an elevated level of mitochondrial oxidative phosphorylation.

There are obvious limitations to the present study. First, we aimed to study changes in mitochondrial bioenergetics in pregnant women in their third trimester of pregnancy, whether diagnosed as healthy or preeclamptic pregnancy. There was a 27-day difference in the mean EGA of each group. However, EGA (or parity) did not significantly impact any of the OCR measurements as factors or as interactions (see Table 2). To control for EGA in comparison groups, a future study would employ longitudinal sampling of a large population of pregnant women beginning early in the third trimester (or earlier), done perhaps once every 2 weeks, given the circulating lifetime of platelets.

Second, we did not attempt to correlate platelet mitochondrial function with that seen in mitochondria harvested at delivery from placental [6–10] or myometrial tissues [11]. A future, more expansive study, harvesting placental or myometrial cells along with platelets from preeclamptic or normal pregnancies, could provide evidence that platelet mitochondria serve as a surrogate for placental or myometrial mitochondria. However, bioenergetic profiling of platelets has been found in other studies to correlate well with differences in brain [31], and skeletal and cardiac [32] tissue bioenergetics.

The final limitation of the study was the heterogeneity of the patient populations. There was no difference in BMI between the two groups of pregnant women. In fact, the relatively large mean BMI reflects our urban population. Obesity is associated with decreased mitochondrial function in mitochondria harvested from muscle cells [33]. We could not find studies showing a similar association in mitochondria harvested from platelets.

As well, though 17 of 20 patients in the preeclamptic group received single or multiple antihypertensive medications (labetalol was the most commonly administered) before the platelet sample was drawn. There was not a rigid protocol for inclusion/exclusion of patients on these medications. Further, though all preeclamptic patients in our study were infused with magnesium sulphate therapy for antiepileptic prophylaxis, only 17 of 20 received magnesium before the study sample was drawn. Therefore, it is possible that some or all of the preeclampsia-associated changes in our study are a consequence of magnesium treatment. While clinical guidelines mandate magnesium sulphate therapy, future studies of platelet bioenergetics profiles should consider blood levels of magnesium as well as the duration of the magnesium therapy before venipuncture.

In the present study, groups were unequal in racial demographics: healthy pregnancy (17 African-Americans); preeclamptic pregnancy (11 African-Americans); non-pregnant (1 African-Americans). There is evidence of racial differences in mtDNA haplogroups of different geographic origin. Platelet mitochondrial bioenergetics from those with an l-haplogroup (maternal African origin) express higher levels for nine mtDNA encoded respiratory complex genes, decreased ATP turnover, and lower levels of ROS production, all consistent with more efficient oxidative phosphorylation [34]. Future studies might investigate the influence of mtDNA haplogroups, platelet mitochondrial respiration and platelet function in normal and preeclamptic pregnancy.

In summary, our results support the use of platelet OCR and ECAR measurements as tools to better understand the potential differences in systemic energy metabolism between non-preeclamptic pregnancy and preeclamptic pregnancy with and without IUGR. We made three striking observations: (i) there is a decline in platelet mitochondrial respiratory function associated with third trimester non-preeclamptic (and otherwise healthy) pregnancy; (ii) this decline is entirely absent from platelets harvested from third trimester pregnancies afflicted with preeclampsia; and (iii) platelets from preeclamptic pregnancies display decreased ECAR, consistent with the possibility of decreased glycolysis and increased oxidative phosphorylation. It remains to be determined whether the reversal of the pregnancy-associated platelet respiratory suppression is relatively unique to preeclampsia or is a feature of multiple disease states. It also remains to be seen whether the pregnancy-linked change in platelet mitochondrial function is adaptive and/or loss of this mechanism in preeclampsia contributes to the disease.

## Clinical perspectives

- Given that mitochondrial bioenergetics abnormalities have been observed in placenta and myometrial cells harvested at delivery of preeclamptic pregnancies, we test the hypothesis that preeclampsia is accompanied by dysfunction of mitochondrial energy metabolism (cellular oxygen consumption and lactate production rates) in circulating maternal platelets during pregnancy.
- The present study found a decline in platelet mitochondrial respiratory function associated with third trimester normal pregnancy prior to 34 weeks’ EGA, a decline absent from platelets harvested in the third trimester pregnancy afflicted with preeclampsia, and a decrease in ECAR in preeclamptic pregnancy consistent with decreased glycolysis and increased oxidative phosphorylation.
- Platelet bioenergetics in preeclampsia are altered compared with those of normal pregnancy suggesting effects of preeclampsia on systemic mitochondrial energy metabolism as well as future studies to confirm circulating platelet bioenergetics as a tool to monitor preeclampsia-associated changes in systemic mitochondrial bioenergetics during pregnancy.

## Abbreviations

BMI: body mass index
ECAR: extracellular acidification rate
EGA: estimated gestational age
HELLP: pregnancy-associated clinical presentation including Hemolysis, Eleveated Liver enzymes and Low Platelet count
IUGR: intrauterine growth restriction
OCR: oxygen consumption rate
RCR: respiratory control ratio
ROS: reactive oxygen species
2,4-DNP: 2,4-dinitrophenol

## References

[1] Williamson R.D., McCarthy C., McCarthy F.P. and Kenny L.C. (2017) Oxidative stress in pre-eclampsia; have we been looking in the wrong place? Pregnancy Hypertens. 8, 1–5 10.1016/j.preghy.2017.01.004 28501272

[2] Ananth C.V., Keyes K.M. and Wapner R.J. (2013) Pre-eclampsia rates in the United States, 1980-2010: age-period-cohort analysis. BMJ 347, f6564 10.1136/bmj.f6564 24201165 PMC3898425

[3] Amaral L.M., Wallace K., Owens M. and LaMarca B. (2017) Pathophysiology and current clinical management of preeclampsia. Curr. Hypertens. Rep. 19, 61 10.1007/s11906-017-0757-7 28689331 PMC5916784

[4] Bernstein P.S., Martin J.N. Jr, Barton J.R., Shields L.E., Druzin M.L., Scavone B.M. (2017) National partnership for maternal safety: consensus bundle on severe hypertension during pregnancy and the postpartum period. Obstet. Gynecol. 130, 347–357 10.1097/AOG.0000000000002115 28697093

[5] Chaiworapongsa T., Chaemsaithong P., Yeo L. and Romero R. (2014) Pre-eclampsia part 1: current understanding of its pathophysiology. Nat. Rev. Nephrol. 10, 466–480 10.1038/nrneph.2014.102 25003615 PMC5893150

[6] Holland O., Dekker N.M., Gallo L.A., Vejzovic M., Fisher J.J. and Perkins A.V. (2017) Review: Placental mitochondrial function and structure in gestational disorders. Placenta 54, 2–9 10.1016/j.placenta.2016.12.012 28024805

[7] Mando C., De P.C., Stampalija T., Anelli G.M., Figus M., Novielli C. (2014) Placental mitochondrial content and function in intrauterine growth restriction and preeclampsia. Am. J. Physiol. Endocrinol. Metab. 306, E404–E413 10.1152/ajpendo.00426.2013 24347055

[8] Maloyan A., Mele J., Muralimanohara B. and Myatt L. (2012) Measurement of mitochondrial respiration in trophoblast culture. Placenta 33, 456–458 10.1016/j.placenta.2012.01.016 22336334 PMC3402169

[9] Wang Y. and Walsh S.W. (1998) Placental mitochondria as a source of oxidative stress in pre-eclampsia. Placenta 19, 581–586 10.1016/S0143-4004(98)90018-2 9859861

[10] Myatt L. (2010) Review: Reactive oxygen and nitrogen species and functional adaptation of the placenta. Placenta 31, S66–S69 10.1016/j.placenta.2009.12.021 20110125 PMC2832707

[11] Vishnyakova P.A., Volodina M.A., Tarasova N.V., Marey M.V., Kan N.E., Khodzhaeva Z.S. (2017) Alterations in antioxidant system, mitochondrial biogenesis and autophagy in preeclamptic myometrium. BBA Clin. 8, 35–42 10.1016/j.bbacli.2017.06.002 28736722 PMC5512187

[12] Leeksma C.H. and Cohen J.A. (1955) Determination of the life of human blood platelets using labelled diisopropylfluorophosphanate. Nature 175, 552–553 10.1038/175552b0 14370167

[13] Zhang W., Siraj S., Zhang R. and Chen Q. (2017) Mitophagy receptor FUNDC1 regulates mitochondrial homeostasis and protects the heart from I/R injury. Autophagy 13, 1080–1081 10.1080/15548627.2017.1300224 28323531 PMC5486361

[14] Kramer P.A., Ravi S., Chacko B., Johnson M.S. and Darley-Usmar V.M. (2014) A review of the mitochondrial and glycolytic metabolism in human platelets and leukocytes: implications for their use as bioenergetic biomarkers. Redox Biol. 2, 206–210 10.1016/j.redox.2013.12.026 24494194 PMC3909784

[15] Chacko B.K., Kramer P.A., Ravi S., Benavides G.A., Mitchell T., Dranka B.P. (2014) The Bioenergetic Health Index: a new concept in mitochondrial translational research. Clin. Sci. (Lond.) 127, 367–373 10.1042/CS20140101 24895057 PMC4202728

[16] Pimentel A.M., Pereira N.R., Costa C.A., Mann G.E., Cordeiro V.S., de Moura R.S. (2013) L-arginine-nitric oxide pathway and oxidative stress in plasma and platelets of patients with pre-eclampsia. Hypertens. Res. 36, 783–788 10.1038/hr.2013.34 23575380

[17] Dranka B.P., Benavides G.A., Diers A.R., Giordano S., Zelickson B.R., Reily C. (2011) Assessing bioenergetic function in response to oxidative stress by metabolic profiling. Free Radic. Biol. Med. 51, 1621–1635 10.1016/j.freeradbiomed.2011.08.005 21872656 PMC3548422

[18] Roberts J.M., August P.A., Bakris G., Barton J.R., Bernstein I.M., Druzin M. (2013) Hypertension in pregnancy. Report of the American College of Obstetricians and Gynecologists’ Task Force on Hypertension in Pregnancy. Obstet. Gynecol. 122, 1122–1131 24150027 10.1097/01.AOG.0000437382.03963.88

[19] Chacko B.K., Kramer P.A., Ravi S., Johnson M.S., Hardy R.W., Ballinger S.W. (2013) Methods for defining distinct bioenergetic profiles in platelets, lymphocytes, monocytes, and neutrophils, and the oxidative burst from human blood. Lab. Invest. 93, 690–700 10.1038/labinvest.2013.53 23528848 PMC3674307

[20] Socol M.L., Weiner C.P., Louis G., Rehnberg K. and Rossi E.C. (1985) Platelet activation in preeclampsia. Am. J. Obstet. Gynecol. 151, 494–497 10.1016/0002-9378(85)90276-5 3156500

[21] Rakoczi I., Tallian F., Bagdany S. and Gati I. (1979) Platelet life-span in normal pregnancy and pre-eclampsia as determined by a non-radioisotope technique. Thromb. Res. 15, 553–556 10.1016/0049-3848(79)90161-0 494160

[22] Gardiner C. and Vatish M. (2017) Impact of haemostatic mechanisms on pathophysiology of preeclampsia. Thromb. Res. 151 (Suppl. 1) , S48–S52 10.1016/S0049-3848(17)30067-1 28262234

[23] Sibai B., Dekker G. and Kupferminc M. (2005) Pre-eclampsia. Lancet 365, 785–799 10.1016/S0140-6736(05)71003-5 15733721

[24] Weinstein L. (2005) Syndrome of hemolysis, elevated liver enzymes, and low platelet count: a severe consequence of hypertension in pregnancy. 1982. Am. J. Obstet. Gynecol. 193, 859 10.1016/j.ajog.2005.02.113 16150287

[25] Kanat-Pektas M., Yesildager U., Tuncer N., Arioz D.T., Nadirgil-Koken G. and Yilmazer M. (2014) Could mean platelet volume in late first trimester of pregnancy predict intrauterine growth restriction and pre-eclampsia? J. Obstet. Gynaecol. Res. 40, 1840–1845 10.1111/jog.12433 25056460

[26] Davies J.R., Fernando R. and Hallworth S.P. (2007) Hemostatic function in healthy pregnant and preeclamptic women: an assessment using the platelet function analyzer (PFA-100) and thromboelastograph. Anesth. Analg. 104, 416–420 10.1213/01.ane.0000253510.00213.05 17242101

[27] Brownfoot F.C., Hastie R., Hannan N.J., Cannon P., Tuohey L., Parry L.J. (2016) Metformin as a prevention and treatment for preeclampsia: effects on soluble fms-like tyrosine kinase 1 and soluble endoglin secretion and endothelial dysfunction. Am. J. Obstet. Gynecol. 214, 356 10.1016/j.ajog.2015.12.019 26721779

[28] Swanepoel A.C. and Pretorius E. (2015) Ultrastructural analysis of platelets during three phases of pregnancy: a qualitative and quantitative investigation. Hematology 20, 39–47 10.1179/1607845413Y.0000000150 24620950

[29] Andreyev A.Y., Kushnareva Y.E., Murphy A.N. and Starkov A.A. (2015) Mitochondrial ROS metabolism: 10 years later. Biochemistry (Mosc.) 80, 517–531 10.1134/S0006297915050028 26071769 PMC4511471

[30] Mookerjee S.A., Goncalves R.L., Gerencser A.A., Nicholls D.G. and Brand M.D. (2015) The contributions of respiration and glycolysis to extracellular acid production. Biochim. Biophys. Acta 1847, 171–181 10.1016/j.bbabio.2014.10.005 25449966

[31] Tyrrell D.J., Bharadwaj M.S., Jorgensen M.J., Register T.C., Shively C., Andrews R.N. (2017) Blood-based bioenergetic profiling reflects differences in brain bioenergetics and metabolism. Oxid. Med. Cell. Longev. 2017, 7317251 10.1155/2017/7317251 29098063 PMC5643153

[32] Tyrrell D.J., Bharadwaj M.S., Jorgensen M.J., Register T.C. and Molina A.J. (2016) Blood cell respirometry is associated with skeletal and cardiac muscle bioenergetics: Implications for a minimally invasive biomarker of mitochondrial health. Redox Biol. 10, 65–77 10.1016/j.redox.2016.09.009 27693859 PMC5045569

[33] Thrush A.B., Dent R., McPherson R. and Harper M.E. (2013) Implications of mitochondrial uncoupling in skeletal muscle in the development and treatment of obesity. FEBS J. 280, 5015–5029 10.1111/febs.12399 23786211

[34] Kenney M.C., Chwa M., Atilano S.R., Falatoonzadeh P., Ramirez C., Malik D. (2014) Molecular and bioenergetic differences between cells with African versus European inherited mitochondrial DNA haplogroups: implications for population susceptibility to diseases. Biochim. Biophys. Acta 1842, 208–219 10.1016/j.bbadis.2013.10.016 24200652 PMC4326177

